# Genomic analysis of a sexually-selected character: EST sequencing and microarray analysis of eye-antennal imaginal discs in the stalk-eyed fly *Teleopsis dalmanni *(Diopsidae)

**DOI:** 10.1186/1471-2164-10-361

**Published:** 2009-08-05

**Authors:** Richard H Baker, Jenna Morgan, Xianhui Wang, Jeffrey L Boore, Gerald S Wilkinson

**Affiliations:** 1 Department of Energy Joint Genome Institute, 2800 Mitchell Drive, Walnut Creek, CA 94598, USA; 2 Sackler Institute for Comparative Genomics, American Museum of Natural History, 79th at Central Park West, New York, New York, 10024, USA; 3 Department of Biology, University of Maryland, College Park, MD 20742, USA; 4 Genome Project Solutions, 1024 Promenade Street, Hercules, CA 94547, USA

## Abstract

**Background:**

Many species of stalk-eyed flies (Diopsidae) possess highly-exaggerated, sexually dimorphic eye-stalks that play an important role in the mating system of these flies. Eye-stalks are increasingly being used as a model system for studying sexual selection, but little is known about the genetic mechanisms producing variation in these ornamental traits. Therefore, we constructed an EST database of genes expressed in the developing eye-antennal imaginal disc of the highly dimorphic species *Teleopsis dalmanni*. We used this set of genes to construct microarray slides and compare patterns of gene expression between lines of flies with divergent eyespan.

**Results:**

We generated 33,229 high-quality ESTs from three non-normalized libraries made from the developing eye-stalk tissue at different developmental stages. EST assembly and annotation produced a total of 7,066 clusters comprising 3,424 unique genes with significant sequence similarity to a protein in either *Drosophila melanogaster *or *Anopheles gambiae*. Comparisons of the transcript profiles at different stages reveal a developmental shift in relative expression from genes involved in anatomical structure formation, transcription, and cell proliferation at the larval stage to genes involved in neurological processes and cuticle production during the pupal stages. Based on alignments of the EST fragments to homologous sequences in *Drosophila *and *Anopheles*, we identified 20 putative gene duplication events in *T. dalmanni *and numerous genes undergoing significantly faster rates of evolution in *T. dalmanni *relative to the other Dipteran species. Microarray experiments identified over 350 genes with significant differential expression between flies from lines selected for high and low relative eyespan but did not reveal any primary biological process or pathway that is driving the expression differences.

**Conclusion:**

The catalogue of genes identified in the EST database provides a valuable framework for a comprehensive examination of the genetic basis of eye-stalk variation. Several candidate genes, such as *crooked legs*, *cdc2*, *CG31917 *and *CG11577*, emerge from the analysis of gene duplication, protein evolution and microarray gene expression. Additional comparisons of expression profiles between, for example, males and females, and species that differ in eye-stalk sexual dimorphism, are now enabled by these resources.

## Background

Sexual selection is responsible for much of the morphological diversity among animals. This diversity is often expressed in the form of male ornamental display traits. Sexually selected characters are unusual among phenotypic traits in that they often maintain high levels of genetic variation despite being influenced by strong selection pressures [1]. This rare combination of abundant intraspecific variation and rapid diversification means that these characters are particularly useful for examining the molecular basis of morphological evolution. Despite widespread study of sexually selected characters, few attempts have been made to identify variation at the molecular level associated with these ornamental traits. Because males and females share most of the same genes, differences in the timing and pattern of gene expression are likely to be the primary mechanism producing sexually dimorphic phenotypes.

Sex is one of the major factors affecting variation in gene expression [2,3]. Studies on Drosophila [4-7] have estimated that between 1/3 and 1/2 of the genome show sex-specific expression, and that much of this variation is heritable [4,8-10]. This variation is mirrored at the interspecific level where even more dramatic patterns of variation have been found. Ranz et al. [7] showed that over 80% of the genes that exhibited expression differences between *D. melanogaster *and *D. simulans *have a sex-specific pattern of change. Half of these evolutionary changes involve the gain, loss or reversal of sex-biased expression and most of these differences involve genes with male-biased expression. However, a subsequent study comparing these taxa did not find such substantial shifts in the pattern of sex-specific gene expression among species [11].

The majority of *Drosophila *microarray experiments examining sex-specific gene expression have focused on whole-body adult tissue. Attempts to separate the sex-specific expression patterns of gonads and somatic tissue [12,13] have found substantially reduced sex-specific expression in somatic tissue relative to gonads. Few studies have examined differential gene expression with respect to a specific sexually dimorphic phenotype. In one example, Barmina et al. [14] used microarray analysis to examine the pattern of gene expression in the developing first and second legs of males and females. The first leg of *D. melanogaster *is sexually dimorphic with respect to bristle patterns while the second leg exhibits little dimorphism. Consistent with the phenotypic differences, they found over 100 genes with sex-specific expression in the first leg but no genes with sexual differences in expression in the second leg. Additional research focusing on the gene expression of specific sexually dimorphic tissue is critical to understanding the genetic factors responsible for the evolution of sex-specific phenotypes. Here, we report on the transcript profile of developing tissue for a highly exaggerated, sexually dimorphic character in the stalk-eyed fly, *Teleopsis dalmanni*.

The elongation of the head into stalks, a condition known as hypercephaly, has evolved independently within Diptera over 20 times [15]. Flies in the family Diopsidae provide one of the most dramatic examples of this phenomenon. In males of some species, the elongation is so extreme that the length of the eye-stalks exceeds the length of the body. Like *Drosophila*, diopsids are part of a subsection of higher Dipterans known as Acalyptrate flies. The Acalyptrata only contain 20% of all described Diptera, but include all of the hypercephalic species. This clustered phylogenetic distribution suggests that Acalyptrate flies may possess some morphological or developmental characteristic that makes them more likely to undergo head modification [15].

There are nearly 200 described diopsid species in 13 genera [16,17]. Comparative analysis has revealed that extremely large, sexually dimorphic, male eyespan has evolved independently within the family several times [18]. Experiments examining the function of eye-stalks in the mating system of diopsids have provided considerable information about their adaptive significance. They are critical as a signaling device in both male-male competition [19,20] and female choice [21,22]. In many sexually dimorphic species, males fight for and defend aggregation sites where matings occur. Both field and lab experiments have demonstrated that the size of a male's eyespan affects his ability to control these mating sites and that females tend to prefer sites controlled by males with larger eye-stalks.

Among diopsids there is substantial variation both between sexes and among species in the amount of heritable genetic variation associated with eyespan [23], and this heritable variation is more tightly linked to overall condition for male eyespan than for other morphological traits [24-26]. In *Teleopsis dalmanni*, artificial selection on male relative eyespan reveals that genes that influence male eyespan also influence female eyespan [27] and exhibit X-linkage [28]. Male eyespan serves as an indicator of genetic quality due to an association between short eyespan and × chromosome segregation distortion [29]. Linkage mapping studies have identified quantitative trait loci (QTL) for eyespan on the X and on both autosomes in males [30]. Overall, quantitative genetic and phylogenetic analyses have demonstrated that the size and variance of eye-stalks is extremely labile, but the genetic mechanisms producing this diversity have not been investigated at the molecular level. Here we provide a catalogue of annotated transcripts present during development of this extraordinary morphological structure.

## Results and discussion

### Functional profile of EST libraries

Three non-normalized cDNA libraries were made from the eye-antennal imaginal discs and optic lobes of *T. dalmanni *at three developmental stages: third instar wandering larva (L), early pupae (P1) and mid pupae (P2). We generated 33,229 high-quality expressed sequence tags (ESTs) from these libraries with 66% of the ESTs coming from the larval library, 10% from the early pupal library and 24% from the mid-pupal library (Table 1). The ESTs are available through NCBI [Genbank:GO271638–GO304866]. The ESTs were assembled into 7,066 clusters containing a total of 11,545 consensus sequences (conseqs). In general, multiple conseqs within a cluster resulted from the presence of non-overlapping sequence, allelic variation or alternative transcripts. A total of 3,697 of the clusters had two or more conseqs and 142 clusters had four or more conseqs. 4,015 clusters contained at least one conseq that had a significant Blast hit (< 1e^-9^) to a protein in *D. melanogaster *and 3,410 unique genes were represented in this list. When blasted against the protein database for *Anopheles gambiae*, 3,296 clusters, comprising 2,847 unique genes, had significant hits. Among the clusters that had a hit to *D. melanogaster*, but not *A. gambiae*, there were 504 unique genes, while 14 clusters had a hit to *A. gambiae *but not *D. melanogaster*. An additional 384 clusters that did not have significant sequence similarity to *D. melanogaster *or *A. gambiae *proteins had significant hits to sequences in the non-redundant protein (nr) and nucleotide (nt) databases with approximately half of these hits being to transposable elements. Fourteen clusters had significant hits to microsatellites previously identified in *T. dalmanni *[31].

**Table 1:** Summary statistics for the sequencing of the eye-antennal cDNA libraries.

Total # of high quality ESTs	33229
Late larval library	21970
Early pupal library	3369
Mid pupal library	7890
# of clusters in assembly	7066
Cluster size distribution:	
# clusters w/ 1 cDNA	4602
# clusters w/ 2–5 cDNAs	1886
# cluster w/ 6–10 cDNAs	403
# clusters w/ >10 cDNAs	175
# clusters w/ significant Blast hits^†^	4029
# of unique genes among Blast hits	3424
# cluster ORFs > 500 bp w/out Blast hit	205

† to *D. melanogaster *and *A. gambiae *protein databases

**Figure 1:**
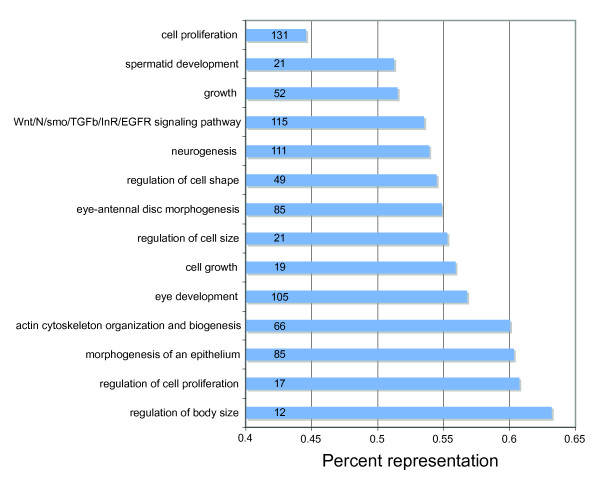
**Percentage of *Drosophila *genes identified in *T. dalmanni *EST database**. For Biological Process (BP) categories that are likely to be important in eye-stalk development and evolution, the bars represent the percentage of *D. melanogaster *genes belonging to that category for which homologues have been identified in the EST database. The numbers within each bar indicate how many genes were found within that category in the *T. dalmanni *libraries.

Because eye-stalks are a complex structure involving modification of the brain, eye, optic nerves and head case, no single GO category is likely to include all, or even most, of the genes involved in eye stalk development. Therefore, we selected from the Gene Ontology database a number of different Biological Process categories, such as cell growth and regulation of cell size and cell shape, that are likely to be important factors in eye-stalk development and calculated for each category the percentage of *D. melanogaster *genes within a given category for which homologues have been identified in the *Teleopsis *EST database (Figure 1). For instance, about 56% of the genes known to affect cell growth in *D. melanogaster *have been identified in the *T. dalmanni *libraries. Overall, we have at least 50% of the genes in most GO categories and 53.6% of all the relevant genes across all categories. This catalogue of genes provides a valuable framework for a comprehensive examination of gene expression in the developing eye disc. In addition, these numbers likely underestimate the percentage of relevant genes discovered in our EST database because the gene lists in *Drosophila *include all genes that are expressed in any developmental stage or participating tissue. Only a subset of these genes is likely to be relevant during eye-antennal disc development.

### Developmental changes in gene expression

**Figure 2:**
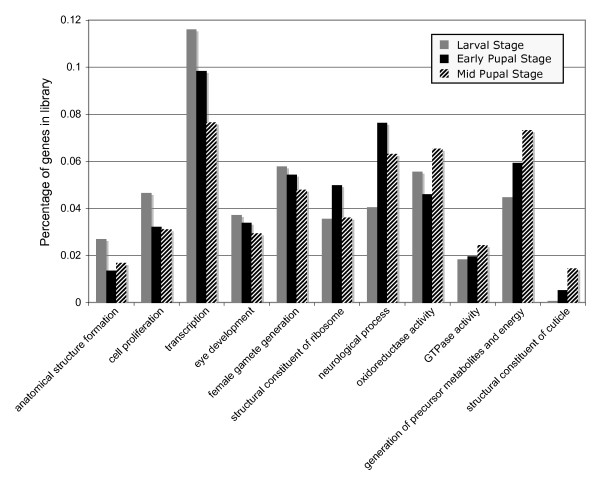
**Gene expression differences among developmental stages**. The percentage of genes in each developmental stage library is presented for GO categories that exhibit significant over-representation in one of the three libraries relative to the EST database as a whole.

Comparison between the EST libraries from the larval, early pupal and mid-pupal developmental stages indicates some significant shifts in the type of genes expressed at each stage. We identified several GO categories that exhibited significant over-representation in one of the libraries relative to the EST database as a whole (Figure 2). In general, the larval stage is characterized by an over-representation of genes involved in anatomical structure formation, transcription, and cell proliferation while the pupal stage exhibits an increase in genes involved in neurogenesis and energy pathways followed by a substantial increase in cuticle production.

Examination of the expression levels for individual genes also reveals substantial differences across libraries as numerous genes are expressed primarily in one developmental stage. Table 2 lists the genes with the largest stage-specific expression patterns as measured by the R-statistic [32]. Thirty additional genes (*Nop56*, *Tenascin major*, *3535983*, *dacapo*, *Heat-shock-protein-70Bb*, *CG7188*, *CG8600*, *Chromatin assembly factor 1 subunit*, *Lk6*, *Baldspot*, *Cyclin A*, *Eukaryotic initiation factor 4B*, *innexin 3*, *Minute (2) 21AB *(3535660), *Protein phosphatase 19C*, *Ribonuclear protein at 97D *(3532397), *Arginine methyltransferase 1*, *CG30015*, *CG32662*, *CG7033*, *Nopp140*, *Tenascin accessory*, *blown fuse*, *Chd64*, *Chloride intracellular channel*, *eyes absent*, *frizzled 2*, *glass*, ORF-141, *Protein disulfide isomerase*) were represented by eight or more cDNAs in the database and were expressed exclusively in the larval library, but had lower R-statistic values due to the disproportionately high EST sampling from this library.

**Table 2:** Abundant, stage-specific transcripts identified in the late larval (L), early pupal (P1) and mid pupal (P2) EST libraries.

		# of cDNAs			
		
Gene Name	R	Total	L stage	P1 stage	P2 stage
CG32603	40.83	66	0	0	66
Arginine kinase	28.33	79	11	1	67
CG30101	19.18	31	0	0	31
Cuticular protein 35B (3531582)	19.18	31	0	0	31
CG34461	16.70	27	0	0	27
Cuticular protein 64Ac	16.70	27	0	0	27
Cuticular protein 35B (3534838)	16.08	26	0	0	26
Osiris 3	14.85	24	0	0	24
Osiris 9	14.23	23	0	0	23
Cuticular protein 92F	9.90	16	0	0	16
CG31203	9.27	19	0	3	16
CG16886 (3534916)	8.66	14	0	0	14
Gasp	8.47	22	2	1	19
CG12163	8.04	13	0	0	13
CG15055	6.80	11	0	0	11
CG17777	6.80	11	0	0	11
Osiris 7	6.80	11	0	0	11
Eukaryotic initiation factor 4a	6.26	90	81	3	6
Misexpression suppressor of KSR 2	6.07	13	1	0	12
ectodermal	6.04	12	0	6	6
CG4409	5.72	11	0	1	10
CG15369	5.49	12	1	0	11
CG2016	5.49	12	1	0	11
CG4962	4.95	8	0	0	8
Cuticular protein 97Ea	4.95	8	0	0	8
Proteasome 26S subunit subunit 4 ATPase	4.83	10	3	7	0
CG1850	4.37	9	0	4	5
Transferrin 1	4.24	9	0	2	7
ATP synthase-beta	4.19	14	3	0	11
cryptocephal	4.08	36	34	0	2
miple	4.01	8	0	1	7
CG18431	3.83	21	21	0	0
Ribosomal protein LP0	3.39	40	36	0	4
short stop	3.28	18	18	0	0
Muscle-specific protein 300	3.09	46	36	8	2
homothorax	3.01	24	23	0	1

In terms of gene families, all representatives of the Enhancer of split complex (*E(spl) mα*, *E(spl) mβ*, *E(spl) m3*, *E(spl) m4*, *E(spl) m7*) identified in the EST libraries were expressed in the larval tissue. In contrast, we identified 11 genes from the Osiris complex (*Osi1*, *Osi3*, *Osi6*, *Osi7*, *Osi9*, *Osi14*, *Osi15*, *Osi18*, *Osi19*, *Osi20*, *Osi22*) and 9 were expressed exclusively in the mid-pupal stage. Little is currently know about the function of the members of this gene family [33], but their strong association with this pupal stage may provide insight about their role in development. Analysis of the GO terms (Figure 2) and gene expression levels (Table 2) indicate that the production of structural cuticle substantially increases during the middle of pupation. In fact, we identified a total of 19 Cuticular proteins (*Cpr30F*, *Cpr35B*, *Cpr49Aa*, *Cpr49Ac*, *Cpr49Ae*, *Cpr51A*, *Cpr56F*, *Cpr57A*, *Cpr62Bb*, *Cpr62Bc*, *Cpr64Ac*, *Cpr65Eb*, *Cpr66Cb*, *Cpr66D*, *Cpr76Bd*, *Cpr92F*, *Cpr97Ea*, *Cpr97Eb*, *Cpr100A*) in the EST database comprising over 200 cDNAs and all but two of them were expressed exclusively in the mid-pupal library.

The distribution of transposable elements within the EST database of *T. dalmanni *exhibited a particularly strong relationship with developmental stage. We blasted (using tblastx) all the conseqs in the EST libraries against the *D. melanogaster *Transposable Element (TE) database and found 191 clusters with significant hits to a total of 71 different types of transposable elements (Table 3). These clusters are more likely to contain reads from the larval library than either of the pupal libraries. Of the 191 clusters with TE hits, 170 are derived exclusively from cDNAs from the larval library, while 10 clusters are exclusive to the early pupal library, 7 clusters from the mid pupal library and 3 clusters from more than one developmental stage. If we look at the total number of cDNAs within these clusters, 252 of the 279 TE cDNAs (90%) come from the larval library whereas only 65% of the total number of cDNAs was sequenced from this library. Furthermore, the mid-pupal library produced 24% of all cDNAs but this stage contains only 4% of the TE reads. This distribution of TE cDNAs across libraries produces an R-statistic = 22.15 and a χ^2 ^= 78.23 (p < 0.0001), indicating that the expression of TEs is significantly biased toward the larval developmental stage.

**Table 3:** Transposable elements identified in the late larval (L), early pupal (P1) and mid pupal (P2) EST libraries.

			# of clusters with TE hit			
			
Transposable Element	Type	Class	Total	L	P1	P2
297	Retroviral	I	1	1	0	0
412	Retroviral	I	2	2	0	0
1731	Retroviral	I	2	2	0	0
3S18	Retroviral	I	2	2	0	0
baggins	non-LTR retrotransposon	I	7	4	1	2
blood	Retroviral	I	2	2	0	0
Burdock	Retroviral	I	1	1	0	0
Cr1a	non-LTR retrotransposon	I	1	1	0	0
Dana\Tom	Retroviral	I	1	1	0	0
Dbuz\Osvaldo	Retroviral	I	1	1	0	0
diver	Retroviral	I	2	2	0	0
diver2	Retroviral	I	4	4	0	0
Dm88	Retroviral	I	4	4	1	0
Dmer\R1A3	non-LTR retrotransposon	I	2	2	0	0
Dmir\TRAM	Retroviral	I	1	1	0	0
Dmir\TRIM	Retroviral	I	3	2	1	0
Doc	non-LTR retrotransposon	I	7	6	1	0
Doc2-element	non-LTR retrotransposon	I	3	3	0	0
Doc3-element	non-LTR retrotransposon	I	3	2	1	0
Doc4-element	non-LTR retrotransposon	I	1	1	0	0
Dsil\Loa	non-LTR retrotransposon	I	4	4	0	0
Dsub\bilbo	non-LTR retrotransposon	I	11	10	1	0
Dvir\Helena	non-LTR retrotransposon	I	1	1	0	0
Dvir\Paris	IR-elements	I	2	1	1	0
Dvir\Penelope	non-LTR retrotransposon	I	5	4	0	1
Dvir\Tel	Retroviral	I	1	1	0	0
F-element	non-LTR retrotransposon	I	8	8	0	0
flea	Retroviral	I	5	5	0	0
Fw2	non-LTR retrotransposon	I	1	0	1	0
G-element	non-LTR retrotransposon	I	1	1	0	0
G2	non-LTR retrotransposon	I	1	1	0	0
G3	non-LTR retrotransposon	I	6	5	0	1
G4	non-LTR retrotransposon	I	10	9	0	1
gtwin	Retroviral	I	2	2	0	0
gypsy4	Retroviral	I	1	1	0	0
gypsy8	Retroviral	I	1	1	0	0
gypsy9	Retroviral	I	1	1	0	0
Helena	non-LTR retrotransposon	I	1	1	0	0
Helitron	Helitron	I	2	2	1	0
HMS-Beagle	Retroviral	I	3	3	0	0
invader3	Retroviral	I	2	2	0	0
invader6	Retroviral	I	1	1	0	0
Ivk	non-LTR retrotransposon	I	1	0	0	1
jockey	non-LTR retrotransposon	I	1	1	0	0
Max-element	Retroviral	I	4	3	1	0
McClintock	Retroviral	I	1	1	0	0
mdg1	Retroviral	I	1	1	0	0
Osvaldo	Retroviral	I	2	2	0	0
Porto1	non-LTR retrotransposon	I	11	10	0	1
R2-element	non-LTR retrotransposon	I	2	2	0	0
roo	Retroviral	I	3	3	0	0
rooA	Retroviral	I	1	1	0	0
rover	Retroviral	I	1	1	0	0
Rt1b	non-LTR retrotransposon	I	2	2	0	0
springer	Retroviral	I	1	1	0	0
Stalker	Retroviral	I	1	1	0	0
TAHRE	non-LTR retrotransposon	I	1	1	0	0
Tirant	Retroviral	I	2	2	0	0
Transpac	Retroviral	I	7	5	1	1
X-element	non-LTR retrotransposon	I	1	1	0	0
ZAM	Retroviral	I	1	1	0	0
Bari1	IR-elements	II	1	1	0	0
Dhet\Uhu	IR-elements	II	4	4	0	0
Dhyd\Minos	IR-elements	II	1	1	0	0
Dmau\mariner	IR-elements	II	14	13	2	1
hobo	IR-elements	II	2	2	0	0
S-element	IR-elements	II	1	1	0	0
S2	IR-elements	II	1	1	0	0
Tc1	IR-elements	II	1	1	0	0
Tc1-2	IR-elements	II	1	1	0	0
Tc3	IR-elements	II	1	1	0	0
						
Total			191	173	13	9

Developmental shifts in the pattern of TE expression have been detected in *Drosophila *[34,35] and there are numerous host factors that can influence the spatial and temporal regulation of TEs [36,37]. Most Class I retrotransposable elements are dependent to some extent on the host transcriptional machinery or specific host transcription factors for their expression [38-41]. The late larval developmental stage in *T. dalmanni *contains an overabundance of genes involved in transcription (Figure 2) that may be influencing the pattern of TE expression either through the presence of specific transcription factors or a general increase in the core transcriptional machinery. However, there is also a disproportionately high number of Class II DNA transposons in the late larval library and these TEs are less likely to be influenced by the dynamics of host transcription activity [36]. An alternative explanation is that mechanisms involved in TE suppression are more active during the pupal stages. Organisms possess a vast array of epigenetic mechanisms that suppress TE activity [37]. One such defense system involves the Argonaute gene family. Proteins from these genes bind to small guide RNAs to form TE silencing complexes [42,43]. *Argonaute-2*, which has been shown to form TE silencing complexes in the somatic tissue of *Drosophila *[43], was identified in the EST database (two cDNA clones from the early pupal library) but not in sufficient quantity to determine if differences in relative distribution exist across developmental stages.

### Paralogous gene assessment

**Figure 3:**
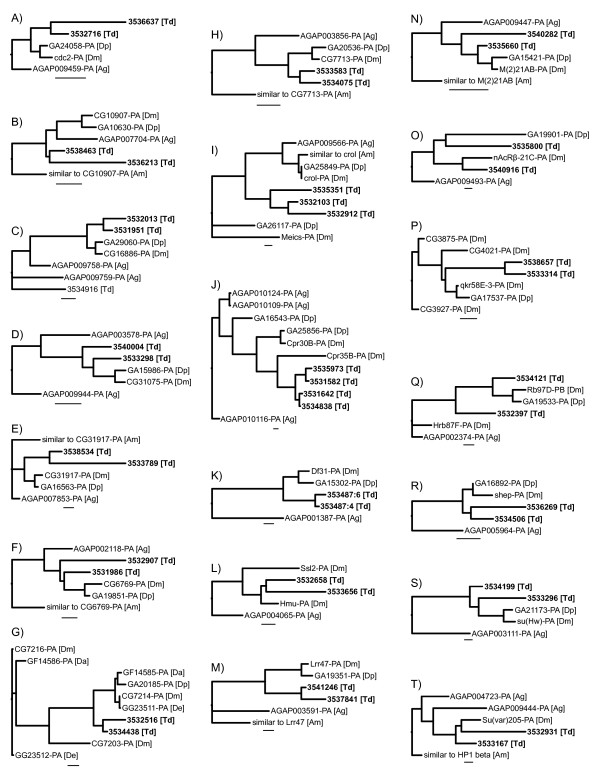
**Putative gene duplication events in the *T. dalmanni *lineage**. A) *cdc2*, B) *CG10907*, C) *CG16886*, D) *CG31075*, E) *CG31917*, F) *CG6769*, G) *CG7214*, H) *CG7713*, I) *crooked legs*, J) *Cuticular protein 35B*, K) *Decondensation factor 31*, L) *Hemomucin*, M) *Leucine-rich repeat 47*, N) *Minute (2) 21AB*, O) *nicotinic acetylcholine receptor beta 21C*, P) *quaking related 58E-3*, Q) *Ribonuclear protein at 97D*, R) *alan shepard*, S) *suppressor of Hairy wing*, T) *Suppressor of variegation 205*. Consensus sequences (conseqs) from different clusters were categorized as paralogous copies of the same gene if the amino acid divergence between the *T. dalmanni *conseqs was greater than 10% and if all conseqs and the top hit gene from *Drosophila melanogaster *are monophyletic relative to the *Anopheles *and *Apis *proteins and other *D. melanogaster *genes. The species included in the phylogenetic analysis are *Anopheles gambiae *(Ag), *Apis mellifera *(Am), *Drosophila ananassae *(Da), *Drosophila erecta *(De), *Drosophila melanogaster *(Dm), *Drosophila pseudoobscura *(Dp), and *Teleopsis dalmanni *(Td). The seven-digit number associated with the *T. dalmanni *clades is the cluster reference number. The scale bar is equivalent to 0.1.

Gene duplication is an important source of novel genetic variation that can facilitate morphological evolution [44-46]. Analysis of EST databases provides a valuable tool for identifying gene duplications or gene expansions particular to the taxon of interest. The central issue in this analysis is to differentiate paralogous gene pairs from allelic variation or alternative transcripts. Here, we have taken a conservative approach and tentatively labeled two or more clusters as paralogous genes established in the diopsid lineage if they meet two criteria: (1) they have greater than 10% amino acid divergence from each other and (2) they are both more closely related in a phylogenetic analysis to one specific *Drosophila *gene than to any other *Drosophila *gene. Using these criteria, we identified 20 pairs or sets of clusters that may have arisen from gene duplication events since the split between *Teleopsis *and *Drosophila *(Figure 3). Alternatively, the duplications may have occurred prior to the *Teleopsis*-*Drosophila *split with subsequent loss of one of the copies before the *Drosophila *radiation.

The branch lengths for the trees in Figure 3 provide some indication of the amount of divergence between the *T. dalmanni *clusters relative to the divergence across Diptera. In 16 of the genes, the divergence between the *T. dalmanni *clusters is greater than that between *D. melanogaster *and *D. pseudoobscura *suggesting that these are true paralogs and not different alleles of the same gene. The lack of monophyly for a number of the cluster pairs may result from the short length of some of the EST amino acid sequence. Sequencing of the entire protein coding region of these genes for *T. dalmanni *and a congeneric taxa will ultimately be necessary to confirm that these clusters are true paralogs and that both copies are functional across the entire length of their sequence. Examination of the gene ontology terms for these putative duplicates indicates that the set of 20 genes is significantly overrepresented for genes involved in spermatogenesis (p = 0.004; *Ribonuclear protein at 97D*, *quaking related 58E-3*, *cdc2*) and mRNA binding (p = 0.005; *suppressor of variegation 205*, *alan shepard*, *Ribonuclear protein at 97D*, *quaking related 58E-3*, *Hemomucin*)

### Protein evolution

Genes that play an important role in the rapid diversification of eye-stalks within diopsids may exhibit rapid rates of change at the protein level. Therefore, we used a measure of relative protein divergence specific to *T. dalmanni *in order to identify genes and GO categories undergoing substantially faster evolution in this lineage. This measure differs from Blast identity percentage because it standardizes the amount of change in the lineage leading to *T. dalmanni *by the total amount of evolutionary change across other flies. Relative divergence is expressed as the percent of total tree length comprised by the branch leading to *T. dalmanni*.

**Figure 4:**
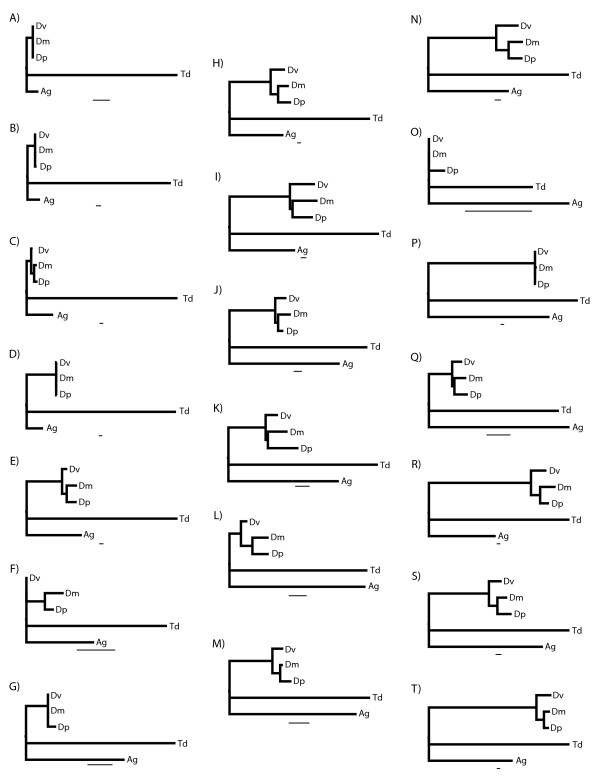
**The twenty fastest evolving genes in *T. dalmanni***. A) *beta-Tubulin at 85D*, B) *CG17293*, C) *rhea*, D) *crooked legs*, E) *CG9520*, F) *ran*, G) *lesswright*, H) *CG4598*, I) *CG6480*, J) *TBPH*, K) *Small ribonucleoprotein particle protein B*, L) *CG31352*, M) *alien*, N) *expanded*, O) *Sec61alpha*, P) *CG31670*, Q) *CG5585*, R) *CNG channel-like*, S) *p115*, T) *CG7372*. Genes were ranked based on the percentage of the total tree length comprised by the branch leading to *T. dalmanni *(Td). The other Dipteran taxa include *A. gambiae *(Ag) and three *Drosophila *species–*D. melanogaster *(Dm), *D. pseudoobscura *(Dp) and *D. virilis *(Dv). Only genes that had at least 150 amino acids of aligned sequence data are shown. All genes exhibited significantly increased rates of change compared to each of the three *Drosophila *species based on a relative rate test. The scale bar is equivalent to 0.02.

Translations of putative protein coding sequence data were obtained for 4,450 contigs comprising 3,230 unique genes with significant homology to a gene in *D. melanogaster*. After alignment to *Drosophila *and *Anopheles *sequences, trimming of poorly aligned sequences and concatenation of multiple non-overlapping fragments, we were left with 2,604 genes for analysis. The gene set contained an average of 342 aligned amino acids (aa) per gene and ranged from 3,673 aa (*shortstop*) to 50 aa (alignments shorter than 50 were excluded from the analysis). Based on a pairwise relative rate test, 72 genes were evolving significantly faster in *T. dalmanni *than in each of the three *Drosophila *species included in the alignment. Figure 4 shows the branch lengths for the 20 most rapidly evolving genes within diopsids that had at least 150 aa in their alignment.

**Figure 5:**
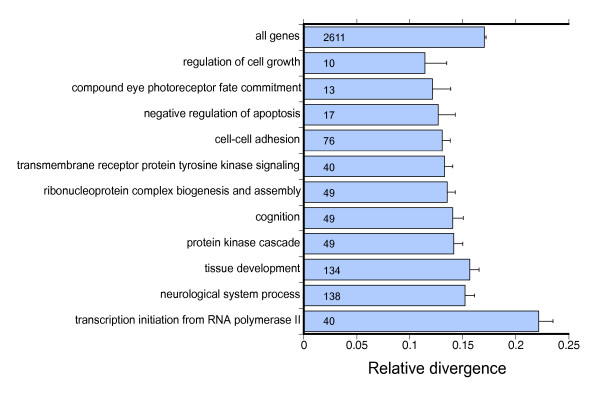
**Biological process categories undergoing significantly slower or faster rates of evolutionary change**. X-axis represents the percentage of the total tree length comprised by the branch leading to *T. dalmanni*. The sample sizes for the various process categories are depicted in the bars. The 'all genes' bar provides the average rate of divergence for all analyzed genes.

Contrary to expectations, functional analysis of the relative gene divergence estimates did not indicate more rapid rates of evolutionary change for genes involved in biological processes expected to be important in eye-stalk development and evolution. Analysis based on the relative divergence rates for all 2,604 genes indicated only a single category, transcription initiation from RNA polymerase II promoter (GO:0006367), was evolving significantly faster than the *T. dalmanni *genes on average (Figure 5). In contrast, several biological process categories–and some, such as neurological system process, compound eye photoreceptor fate commitment and regulation of cell growth, that are likely to be important in eye-stalk development–are evolving significantly slower than expected (Figure 5). Overall, when we combine all the genes that fall within the 'important eye-stalk' BP categories listed in Figure 1, these genes are evolving slightly slower, but not significantly different, than the rest of the genes (P = 0.099, t = -1.65).

The numerous slow-evolving categories that characterize the *T. dalmanni *lineage may result from strong stabilizing selection acting on genes in these categories in general. If positive selection is operating on these biological processes at all it may be limited to only a few genes in each category. For instance, the gene *crooked legs *(*crol*) is involved in two of the slow-evolving categories–cell adhesion and tissue development–and, recently, has been shown to represent an important step in the pathway linking ecdysone signaling and cell proliferation [47]. Unlike other genes involved in these BP categories, *crol *is also undergoing extremely rapid protein evolution (Figure 4) and appears to have duplicated at least twice within the lineage leading to *T. dalmanni *(Figure 3). Alternatively, the gene set characterized by the list of 'important eye-stalk' BP categories may not reflect the processes that are actually important in eye-stalk evolution or rates of protein evolution may not provide a valuable indicator of their relevance to eye-stalk evolution. It is also important to note that, because these estimates of gene divergence are derived from ESTs that represent only partial gene fragments, the estimated rates of evolution may be different when the entire protein coding sequence is evaluated. Furthermore, additional sampling of species within Diptera is necessary to verify that apparent rate differences between *T. dalmanni *and *Drosophila *are actually specific to diopsid lineages.

### Divergence in gene expression between selection lines

Using the EST sequences to construct probes for oligonucleotide microarrays, we conducted an experiment examining differences in gene expression between lines of flies selected for longer or shorter relative eyespan. Analysis of eight replicate arrays [Genbank:GSE15444] revealed that 367 of 3,105 genes exhibit differential expression based on a false discovery rate of 1%. The d-statistics for the significant genes were either greater than 2.26 or less than -2.26. Of these genes, 44 had d-statistics exceeding 5 (Table 4), 27 exhibited more than a two-fold difference in expression between the lines, and one gene, *CG11577*, exhibited 8 times greater expression in flies from the most extreme high line than from the most extreme low line. Among the 367 significant genes, 40 exhibited no detectable homology to any gene in the *Drosophila *database, and 50 belonged to the set of 'important eye-stalk' genes defined in Figure 1. Fourteen of the differentially expressed genes (by *S6*, *cdc2*, *CG10283*, *CG1575*, *CG15835*, *CG31917*, *CG4598*, *CG6480*, *Kruppel*, *lesswright*, *lethal (2) k14505*, *par-6*, *Ribonucleoside diphosphate reductase small subunit*, *Small ribonucleoprotein particle protein B*) are also evolving significantly faster in *T. dalmanni *than other flies and, of these, 2 genes (*cdc2 *and *CG31917*) have duplicates in the EST database.

**Table 4:** Differentially expressed genes between flies that have been bred for increased and decreased eyespan.

Gene^‡^	FlyBase ID	Ave LR^†^	d-statistic
X-22	NA	1.953	14.512
CTCF	FBgn0035769	1.861	11.672
CG11577	FBgn0036847	3.012	11.665
ORF-102	NA	1.261	10.038
Lamin	FBgn0002525	-1.393	-9.444
Ornithine aminotransferase precursor	FBgn0022774	-1.571	-8.991
parcas	FBgn0033988	-1.256	-8.768
visceral mesodermal armadillo-repeats	FBgn0022960	-1.266	-8.7
CG11409	FBgn0024366	1.25	8.517
CG30390	FBgn0050390	-1.284	-8.479
CG4908	FBgn0032195	1.052	8.291
eIF3-S8	FBgn0034258	-1.573	-8.279
prominin-like	FBgn0026189	1.307	7.912
AGAP005746	NA	0.881	7.723
Aut1	FBgn0036813	0.867	7.61
ballchen	FBgn0027889	1.007	7.364
ORF-79	NA	-1.205	-7.257
CG11309	FBgn0037070	-0.843	-7.174
M-spondin	FBgn0020269	1.136	6.935
X-15	NA	-1.609	-6.807
Baldspot	FBgn0036650	-1.198	-6.795
embryonic lethal, abnormal vision	FBgn0000570	1.027	6.498
Ecdysone-inducible gene L2	FBgn0001257	-0.807	-6.346
X-20	NA	1.101	6.094
cdc2 (3536637) 3536637:2	FBgn0004106	0.875	6.056
Small ribonucleoprotein particle protein B	FBgn0010083	-0.709	-6.015
CG4858	FBgn0037011	-0.623	-5.959
ORF-44	NA	-0.728	-5.906
phantom	FBgn0004959	0.942	5.892
yellow-c	FBgn0041713	0.854	5.882
CG4680	FBgn0036627	-1.25	-5.824
CG13025	FBgn0036660	1.159	5.652
Clathrin heavy chain	FBgn0000319	0.915	5.641
CG31665	FBgn0051665	0.893	5.528
Succinyl coenzyme A synthetase alpha subunit	FBgn0004888	0.718	5.487
CG7077	FBgn0038946	0.919	5.456
bendless	FBgn0000173	0.791	5.267
CG7861	FBgn0033055	1.036	5.214
X-68	NA	-0.798	-5.197
CG17510	FBgn0039969	-0.636	-5.173
CG40500	FBgn0069968	0.937	5.151
CG14911	FBgn0035701	0.825	5.136
dim gamma-tubulin 6	FBgn0039638	0.766	5.13
CG30104	FBgn0050104	0.681	5.066

† Positive log ratios (LR) indicate genes that are expressed at increased levels in the high line flies. ‡ Genes that begin with 'ORF-' or 'X-' do not have homologous sequences in *D. melanogaster*. 'ORF-' genes have an open reading frame larger than 300 bp while 'X-' do not. See Methods for details on d-statistic.

Functional analyses of the microarray results do not reveal a strong pattern of relationships among the set of 367 significant differentially expressed genes. GeneMerge did not identify any GO categories with significant overrepresentation when all differentially expressed genes were included in the analysis set. When only genes that were significantly down-regulated (i.e. had higher expression values in the low line flies) were analyzed, genes involved in RNA splicing factor activity, transesterification mechanism (GO:0031202) were significantly overrepresented (P = 0.0362). No significant GO categories were found among the set of up-regulated genes.

We also tested for differences in the mean d-statistic for each biological process category relative to all the genes. This functional assay utilizes the expression values for all of the genes, not just those with significant expression difference between lines. Based on this analysis, genes that play a role in gamete generation (P < 0.001), embryonic development (P < 0.001), cell-cell adhesion (P = 0.015) and neurological system process (P = 0.03) have significantly higher mean d-statistics than an average gene set of similar size. This result indicates that the high line flies had higher expression values for genes in these GO categories than the low line flies. It is likely that this pattern results from a shift in either the developmental timing or allometric relationship among body parts between lines. For instance, genes involved in embryonic development are expressed relatively early in the metamorphic process compared with other genes in the EST database, so if high line flies have delayed their differentiation to allow for extra imaginal disc growth relative to low line flies, an increase in the expression of embryonic development genes might occur. Consistent with this interpretation, flies from the high lines exhibit a steeper allometric relationship between eyespan and body length [27] and take longer to develop [48] than flies from the low lines. Similarly, the optic nerve may represent a larger proportion of the overall tissue in high line flies than in low line flies, which could result in a slight increase in the overall level of gene expression for neurological system process genes. Distinguishing between these possibilities will require additional experiments in which gene expression between replicate and control lines is compared at multiple time points during development.

### Quantitative rtPCR

**Figure 6:**
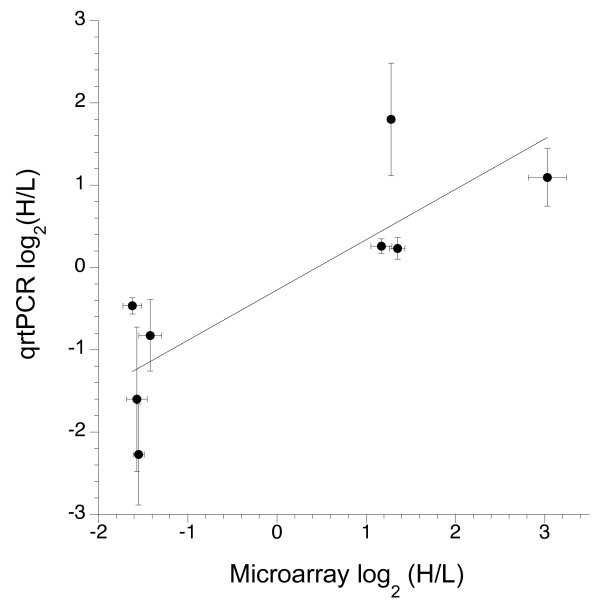
**Relative gene expression estimated by oligoarrays correlates with relative expression estimated by qrtPCR**. Calculation of fold change for eight target genes was estimated relative to the expression level of a control gene, GAPDH, using the 2^-ΔΔCT ^method. Error bars indicate one standard error and are based on four biological replicates for each selected line for qrtPCR and eight biological replicates for microarrays.

Relative expression of eight genes was estimated using quantitative reverse transcription PCR (qrtPCR). The correlation between the average relative expression detected by the microarray and by qrtPCR was 0.83 (Figure 6). Variation was considerably greater for qrtPCR estimates because four, rather than eight, replicates were performed. Nevertheless, all four genes with greater high than low line expression showed increased high line expression by qrtPCR and the four genes with greater low than high line expression showed increased low line expression by qrtPCR.

## Conclusion

Stalk-eyed flies in the family Diopsidae provide an excellent model system for studying sexual selection. They possess a highly exaggerated ornamental character that plays an essential role in the mating system of numerous species and exhibits abundant intraspecific and interspecific variation. Elevated levels of heritable genetic variation is a common feature of sexually selected traits [1] and has been demonstrated in the eye-stalks of diopsids [23]. However, the molecular basis of this genetic variation is not well understood in any system. Developing a comprehensive and well-annotated catalogue of genes expressed in the tissue developing into ornamental morphologies is a critical step in this process. Overall, an understanding of the genetic architecture controlling eye-stalk development and evolution will provide critical insights concerning 1) the developmental mechanisms controlling allometry, 2) the genetic basis of sexual dimorphism and the convergent evolution of dimorphism and 3) the evolution of condition dependence of ornamental traits. Here, we report on the transcript profile of the eye-antennal imaginal disc of the sexually dimorphic stalk-eyed fly *Teleopsis dalmanni*. This EST database will provide a valuable foundation for further investigations into the genetic and molecular basis of eye-stalk variation.

Variation in gene expression that shapes morphological diversity is likely to be tissue and time dependent [14]. By focusing our analysis of gene expression specifically within the eye-antennal discs and adjacent optic lobes at three developmental stages we are able to better isolate genes important in the development and evolution of eye-stalks. A microarray experiment examined differences in gene expression between lines of flies that have been artificially selected for divergent eyespan. While this experiment did not directly examine differences in gene expression between males and females, the selection lines do differ in their extent of eye-stalk sexual dimorphism. Numerous genes exhibited significantly divergent levels of expression between selection lines but no obvious causal genetic mechanism or pathway emerged from functional analysis of these genes. The evolution of eye-stalk and sexual dimorphism in eye-stalks is undoubtedly a complicated morphological innovation involving numerous genes and pathways, so it is not surprising that a single gene expression comparison failed to pinpoint a primary genetic basis of eye-stalk variation. Additional microarray experiments are currently underway to quantify sex-biased expression in *T. dalmanni *and a congeneric monomorphic species, *T. quinqueguttata*.

Despite a lack of clear signal when analyzed across pathways or higher level biological processes, analysis of gene expression across the EST libraries and microarray experiment revealed several candidate genes that may play an important role in eye-stalk evolution. Some of the more noteworthy genes include *crol*, which has undergone at least two duplications and rapid protein evolution in diopsids, *CG11577*, which showed an 8-fold expression difference between selection lines and *cdc2 *and *CG31917 *which both exhibit duplicate copies, rapid protein evolution and differential gene expression between selection line flies. Little is known about the function of *CG11577 *and *CG31917 *in *Drosophila*, but both *crol *and *cdc2 *are important developmental genes influencing cell cycle progression [47,49]. *Crol *is of particular interest because it is regulated by ecdysone and mutations in the gene influence head eversion and appendage elongation [50,51], phenotypic effects that are likely to be relevant to eye-stalk development.

## Methods

### cDNA library construction and EST sequencing

The adult head structures of Acalyptrate flies derive from a pair of cell primordia known as the eye-antennal imaginal discs. These discs undergo dramatic growth and differentiation during larval and pupal stages, transforming from a flat sheet of relatively few cells into the eyes, antenna and head capsule of adult flies. Histological [52] and fate mapping studies [53] indicate that eye-stalk development and growth begins in the late larval stage and continues during pupal development.

Larvae and pupae were harvested from a large outbred laboratory population of *Teleopsis dalmanni *that was originally collected near the village of Ulu Gombak in peninsular Malaysia in 1999 [54]. Larvae were reared at low density in 50 ml of pureed corn and kept in an incubator at 25°C with a 12 h L:D cycle. Eye-antennal discs and optic lobes were dissected from approximately 200 flies at each of three stages and stored in RNAlater (Ambion) at -20°C.

Libraries were made from three developmental stages – late larval flies (L), early pupal flies (P1) and mid-pupal flies (P2). The larval period was defined as the interval in which third instar larvae stop feeding, purge their gut and wander toward a suitable pupation location, early pupation was defined as 0–3 days after initiation of pupation and mid-pupation was defined as 4–6 days after initiation of pupation. Total RNA was prepared using an RNA isolation kit (Promega) and RNA quality was verified using an Agilent bioanalyzer. We then synthesized ds cDNA using the SMART PCR cDNA synthesis kit (Clontech). The libraries were constructed following the manufacturer's protocol except that two different ligation reactions were performed on size-selected samples. The total cDNA sample was run on an agarose gel and cut into two fragments corresponding to transcripts above and below 1 kilobase. These fragments were then purified, precipitated and ligated into the vector separately. This procedure ensured that the library did not contain a majority of small inserts that preferentially ligate into the vector. Averaged across the three developmental stages, the small insert libraries had an average insert size of 600 bp and 3.5% of clones were without an insert, while the large insert libraries had an average insert size of 1.25 kb and 15% of clones were without an insert.

Cloned cDNAs were transformed into ElectroMAX DH10B cells (Invitrogen), and, after growth, the colonies were picked from bioassay plates using a Q-bot automated colony picker (Genetix) and stored individually in 384-well plates. We sequenced a total of 24,192 cDNAs (14,976 from the larval library, 3,072 from the early pupal library and 6,144 from the late pupal library) in both the 5' and 3' directions. Preliminary assessment of library quality indicated that the larval library had fewer clones without inserts and higher complexity than the other two libraries, so the majority of ESTs were sequenced from this library. Sequencing reactions followed standard JGI protocol  using rolling circle amplification with TempliPhi ET terminators (Amersham), SPRI reaction clean up, and electorophoretic separation on either a Megabace 4000 or ABI 3730 × l automated DNA sequencer.

### EST annotation

Clustering of EST reads into consensus sequences was performed by the Joint Genome Institute's EST Analysis Pipeline (see Additional file 1). Base assignment and quality scores were determined using Phred software [55,56]. Vector, linker, adapter, poly-A/T and other artifact sequences were removed using the Cross_match software (available with the Phrap package), and an internally developed short pattern finder. ESTs shorter than 100 bp were removed from the data set, as were contaminant sequences such as *E. coli*, common vectors and sequencing standards. Clustering of the remaining ESTs was conducted using a two-step process. In the first step, pairwise EST alignments were generated using a modified version of the Smith-Waterman algorithm [57], which was developed at the JGI for use in whole genome shotgun assembly. ESTs with 96% sequence similarity over a span of 100 bp were included in the same cluster. The 5' and 3' reads from the same cDNA clone were also combined into the same cluster. In the second step, all reads within a given cluster were assembled into consensus sequences using Phrap [58]. Many clusters contained more than one consensus sequence (referred to as conseqs within the text). Alternative consensus sequences within a given cluster typically represented 1) non-overlapping regions of the same gene, 2) transcript variants of the same gene or 3) allelic variants of the same gene. Because most conseqs from a given cluster belonged to the same gene, the functional annotation of the ESTs was organized primarily at the level of cluster.

All conseqs from the EST database were blasted (using blastx with a cut-off of 1 × e^-9^) against the protein databases for *Drosophila melanogaster *(release 5.6 from FlyBase) and *Anopheles gambiae *(release AgamP3.49 from Ensembl) as well as the Genbank non-redundant DNA and protein databases (nr). Because of the close phylogenetic proximity of *T. dalmanni *to *Drosophila*, the *D. melanogaster *protein and gene information was used as the default reference for all *T. dalmanni *annotation (see Additional file 2). All cases in which two conseqs from the same cluster had different *D. melanogaster *genes as their top hit were resolved by examining the e-values of the secondary Blast hits for each conseq. Twenty-seven clusters contained conseqs that appeared to belong to different genes and these conseqs were separated in subsequent annotation. We also blasted (using tblastx) all conseqs against the *D. melanogaster *Transposable Element database (version 9.4.1). The largest open reading frame for each conseq was generated using the GetOrf module in the Mobyle analysis portal [59].

Gene Ontology annotations for the library as a whole and for each developmental stage were constructed and compared to each other and to *D. melanogaster *using GeneMerge [60]. All of the ESTs from a given developmental stage library were not assembled and annotated separately. Rather, the ESTs for all three libraries were assembled and annotated together and a gene was assigned to a given developmental stage if a cluster that had a top hit to that gene contained a sequencing read from at least one cDNA belonging to that developmental stage library. We also constructed a list of all genes whose representation in the EST database was unique to a particular developmental stage. Using GeneMerge, we tested whether genes belonging to any given GO category were overrepresented in a particular developmental stage relative to the EST database as a whole. Representative GO terms were chosen to summarize a single biological process pathway if several of the terms in a parent/child relationship were significant.

### Paralogous genes assessment

In numerous cases, different clusters contained conseqs whose top Blast hit was to overlapping regions of the same *D. melanogaster *gene. These clusters were candidates for paralogous gene sets, possibly arising from gene duplication since the lineage split that led to *D. melanogaster*. To test this possibility, we aligned all the conseqs with this condition (a total of 1,389 clusters representing 329 genes) and visually inspected the alignments to determine why the sequences had not been combined into the same cluster if they were homologous to the same region in *D. melanogaster*. For the majority of genes, the hypothesis of paralogous gene pairs or sets could be rejected by visual inspection. Common reasons for their separation into different clusters included 1) mis-splicing (one of the cluster conseqs retained an intron), 2) alternative transcripts (sequence similarity broke down at an exon border), 3) large indels, and 4) UTR variation. For the remaining genes that showed widespread nucleotide variation across the protein coding sequence, conseqs were blasted (using blastx) against the insect genome database in GenBank to identify homologous transcripts in *D. melanogaster*, *D. pseudoobscura*, *A. gambiae *and *Apis mellifera*. In one case, no homologous protein could be identified in *A. gambiae *or *A. mellifera *so we included additional *Drosophila *species in the analysis. All genes within e^-20 ^of the top hit were retained and aligned with the translations for the *T. dalmanni *conseqs using ClustalX [61]. Based on these alignments, our criteria for calling a pair or set of conseqs from different clusters distinct genes was if the amino acid divergence across homologous regions of the *T. dalmanni *conseqs was greater than 10% and if all conseqs and the top hit gene from *D. melanogaster *are monophyletic relative to the *Anopheles *and *Apis *proteins and other *D. melanogaster *genes. The phylogenetic relationships among the proteins were determined using the proml module in the Phylip v3.68 software package [62] with a JTT model of change and no rate variation among sites.

### Protein evolution

Identifying genes evolving rapidly in the lineage leading to *T. dalmanni *relative to their rate of change among other Dipteran species may provide insight about possible mechanisms important in the evolution of eye-stalks. Therefore, we constructed a database of all unique protein coding data among the *T. dalmanni *EST sequences to align with amino acid sequences from *Anopheles *and *Drosophila*. All conseqs belonging to a cluster with a significant Blast hit to a *Drosophila *gene were aligned in Sequencher v. 4 (Gene Codes) using the Large Gap assembly algorithm and a 90% match minimum. All contigs were visually inspected to ensure proper homology and the consensus sequences were blasted (using blastx) against the *D. melanogaster *protein database. These blast results identified possible frame shifts and intron inclusions because they appear as multiple alignments in the Blast output. For all of these cases, the sequencher contigs were inspected to confirm the presence of a frame shift or intron and the affected assemblies were fixed. All open reading frames larger than 200 bp for each contig were then generated with GetOrf [59]. The translations for these ORFs were blasted against the *D. melanogaster *protein database to identify which ORF was the true protein coding sequence (in some cases the largest ORF of a conseq was not the one with homology to the *Drosophila *gene). In a few cases, two ORFs from a single conseq had significant hits to different *D. melanogaster *gene. This resulted from the genes sharing UTR sequence (in the majority of cases the genes are adjacent in *D. melanogaster*) and, therefore, being clustered together during assembly. Both ORFs were retained for analysis.

Overall, this methodology produced a total of 4,450 unique, non-overlapping protein sequences corresponding to 3,230 genes in *D. melanogaster*. All protein sequences were then aligned to homologous regions in *A. gambiae*, *D. melanogaster*, *D. pseudoobscura and D. virilis*. The three *Drosophila *species were chosen to span the full range of evolutionary relationships within the genus. Homologous protein sequences in *Drosophila *were obtained from the 12 Drosophila species analysis ftp site  and from Ensembl BioMart for the *A. gambiae *homologous proteins.

Each of the 4,450 unique *T. dalmanni *protein sequences was aligned to its *D. melanogaster *homolog using JAligner, which implements the Smith-Waterman [57] pairwise local alignment algorithm with Gotoh's [63] improvement. Columns containing leading and trailing gaps in the *T. dalmanni *sequence were removed from the alignment. Poorly aligned regions at each end were further trimmed using an alignment quality score method described in Wu et al. [64], and modified for amino acid sequences by incorporating the BLOSUM62 matrix. A multiple sequence alignment was then built using HMMER v2.3.2 [65], aligning the homologous sequences in *T. dalmanni, A. gambiae*, *D. melanogaster*, *D. pseudoobscura *(or *D. persimillis*) *and D. virilis *(or *D. mojavensis*) to each profile HMM. Multiple protein sequences that correspond to a single gene in *D. melanogaster *were concatenated prior to further analysis. Alignments are provided as supplemental material (see Additional file 3). Phylogenetic trees were constructed using the PhyML software package [66] using PhyML with the JTT substitution model with no invariant sites, constraining the topology to be ((Dv,(Dm, Dp)), Td, Ag), and optimizing branch lengths and rate parameters.

To estimate the rate of relative divergence in the *T. dalmanni *lineage relative to the other dipteran species, we calculated the percentage of the entire tree length comprised by the branch leading to *T. dalmanni *(see Additional file 4). Thus, the higher the percentage, the faster that gene was evolving in the lineage leading to *T. dalmanni *relative to the *Drosophila *and *A. gambiae *lineages. This statistic was used as input data in the program ErmineJ [67] to determine if genes belonging to a given Biological Process category are evolving significantly faster or slower than expected based on the entire distribution of relative branch lengths for all genes in the EST database. This program uses a resampling technique in which the mean values for a given statistic (*T. dalmanni *relative branch lengths in this study) for each Biological Process category is compared to a distribution of resampled mean values and uses a Benjamini-Hochberg correction of p-values. We implemented the 'Gene score resampling' option, with a negative log transformation for all Biological Process categories that had more than 10 and less than 200 representatives. We also conducted pairwise relative rate tests on individual genes as implemented in HyPhy [68].

### Microarray Analysis

#### Slide Construction

Microarray slides were constructed for all clusters in the EST database that had 1) significant sequence identity to known genes in *Drosophila *or *Anopheles*, 2) an ORF larger than 300 bp or 3) more than four cDNA clones associated with that cluster. Using Agilent software, we designed three 60-mer oligonucleotides for each gene based on the entire EST sequence for the conseq with the highest Blast hit or largest ORF within each cluster. We then had slides with four 44,000-feature arrays containing this probe group synthesized by Agilent. Each probe was spotted in triplicate on the slide.

#### Hybridization and analysis of expression

As a first step towards identifying genes that influence relative eye-stalk length we compared gene expression between lines of flies that have been under artificial sexual selection to increase or decrease male eye span for 65 generations [27,28,30]. We compared gene expression between male flies from the high (H2) and low (L2) lines that deviated furthest from the average control lines. At the time of these experiments H2 male eyespan was 9.36 ± 0.07 mm and L2 male eyespan was 7.39 ± 0.07 mm, which represents a difference of 5.7 s.d. between these two lines.

To minimize developmental variation in gene expression we collected tissue from wandering larvae just prior to pupation. Two lines of evidence indicate that eye-stalk development begins at this time. By staining larval and pupal sections, Buschbeck et al. [52] showed that the eye discs begin rapid cell growth during the pre-pupal period. In addition, application of synthetic juvenile hormone (methoprene) to pre-pupal flies caused a significant shift in male eyespan to body length allometry in adult flies [69], indicating that eye-stalk expression can be influenced by hormone titers in wandering larvae. Therefore, we collected imaginal disc and brain tissue from wandering larvae just after gut purge. To recognize larvae without food in their digestive tract we reared larvae on food that had been dyed with green food color. Using this technique, gut-purged larvae have transparent, instead of green, digestive tracts.

Larvae were dissected in phosphate buffered saline (PBS) and the larval cuticle was inverted from each end to expose genital and eye discs. Male larvae were identified by genital disc morphology and their eye discs, optic lobes and brains were removed and submerged in RNAlater solution (Ambion) and stored at -20°. Total RNA was extracted from disc and brain tissue using an RNA isolation kit (Promega). Each biological sample consisted of twenty-five sets of eye discs that were homogenized by grinding with plastic pestles in 175 μl lysis buffer. After treating with DNase I enzyme, RNA was concentrated by lyophilization and the quality of each sample was checked with an Agilent 2100 bioanalyzer. Only high-quality RNA samples (RNA integrity above 7) were selected for the experiments. A total of eight pairs of samples were used in this experiment.

We used the Ovation amino allyl RNA amplification kit (NuGen) to amplify messenger RNA prior to labeling. This procedure uses reverse transcription to make single-stranded cDNA and then DNA polymerization to make double-stranded (ds) cDNA. The ds cDNA is then amplified using a linear amplification procedure. Samples were alternately labeled with Cy3 and Cy5 dyes using an amino allyl cDNA labeling kit (Ambion) with regard to source of selection line and then hybridized to an oligoarray using an Agilent rotator rack and oven. After hybridization, slides were scanned at 5 μm resolution using a Genepix 4200A scanner.

Gene expression was measured from array images scanned at each dye wavelength using the commercial software program, Genepix Pro (see Additional file 5). Features were excluded from further analysis if the majority of pixels were saturated, the spot was noncircular, or if the squared correlation between channels was less than 0.3. Median intensity ratios were normalized to remove dye bias without subtracting background using MIDAS software developed by The Institute for Genome Research (TIGR). Intensity ratios for dye-swapped samples were inverted and the hypothesis of no differential expression was tested for each gene by SAM [70] using MeV software from TIGR. Genes were excluded from testing if data from fewer than four arrays were available for analysis, which left 3,105 genes. We analyzed the data in two ways: using the log-ratio expression intensity for each oligo as an independent observation and using the average log-ratio of all oligos for each gene. Many of the same genes were identified as being differentially expressed by both methods, so we report only the results from the average oligo analysis. With 8 replicate arrays, significance for each gene is assessed using 256 permutations. Significance was assigned for any gene with a q-value of 0 [71]. We used the d-statistic, which measures the standardized difference between the mean log-ratios for the eight replicate arrays and zero, to rank order the degree of bias in expression for each gene. The microarray data discussed in this publication have been deposited in NCBI's Gene Expression Omnibus and are accessible through GEO Series accession number GSE15444.

Functional analysis of the log-ratios was conducted using GeneMerge [60] and ErmineJ [67]. With GeneMerge, the set of genes showing significant differential expression (either in total or just the up-regulated and down-regulated sets) was compared to all the genes on the microarray slide to identify GO categories with significant over-representation within the set of differentially expressed genes. With ErmineJ, the d-statistic for all the genes with detectable expression values was converted to a measure varying between 0 and 1 and used as input to determine if any GO categories had significantly larger or smaller mean d-statistic values than an average gene set of similar size. In this program, we implemented the 'Gene score resampling' option, with a negative log transformation for all Biological Process categories that had more than 10 and less than 200 representatives.

### Quantitative rtPCR

To validate the microarray results we used quantitative reverse transcriptase PCR (qrtPCR) to quantify relative expression of eight genes identified as exhibiting differential expression. The eight genes were *Baldspot, CG11577*, *CG30390*, *Lamin, M-spondin*, ORF-102, *Ornithine aminotransferase precursor*, and *prominin-like*. Primer design and optimization was done with Beacon Designer 7.0 software (Baldspot: 5'-ACTTCAGTTACTTTGTGCTATTCG-3', 5'-CTCCGCCGCCATTTGC-3'; CG11577: 5'-GTGGTGCTGAGGAGAAAGAAG-3', 5'-ATTCACTGCGTCGGTATTCG-3'; CG30390: 5'-GCTTATGCGGATGAAACAC-3', 5'-TCAGATGGCTATACAGAACC-3'; Lamin: 5'-CGACAAGGTCTAAGCGTTC-3', 5'-GGCGAGTTGGACTTAATGG-3'; M-spondin: 5'-CAATAAATGGTGTTGGCAATCAGC-3', 5'-TTCTCCGTTCGCCGTGTTG-3'; ORF-102: 5'-TTGGCTCGCAGACATCTACC-3', 5'-CGTCCGTGGGCATAAGGG-3'; Ornithine aminotransferase precursor: 5'-ATTTCTGGGGACGCACATTATCG-3', 5'-CCTGGCATGAATGGACCGAATC-3'; prominin-like: 5'-CATCAGTGCCGCTGTCAAC-3', 5'-ATTTCTCGGTTTCCTGCTCATAC-3'). The annealing temperature of RT-PCR was optimized in a gradient cycler. First stand cDNA synthesis was performed using 0.5 μg of total RNA and Superscript II reverse transcriptase (Invitrogen) in 20 μl according to the manufacturer's instructions. The product of reverse transcription was diluted 5 fold for PCR. Real-time quantitative PCR was performed using a Lightcycler 480 (Roche Molecular Biochemicals) and SYBR Green I. Briefly, 1 μl of first stand cDNA reaction was amplified by each primer pair in a 20 μl reaction containing 7 μl water, 1 μl forward primer (10 μM), 1 μl backward primer (10 μM), 10 μl Lightcycler DNA Master Mix SYBR Green I (2×) (Roche). The amplification reaction consisted of 45 cycles of denaturation at 94°C (10 seconds), annealing at 60°C (10 seconds), and extension at 70°C (10 seconds). Fluorescent measurements were obtained once in each cycle by sequential fluorescence monitoring of each sample tube at the end of extension. A fractional cycle number or crossing point (CP) was determined from the exponential phase of the fluorescence amplification profiles using the second derivative maximum function of the Roche Lightcycler 480 basic software. Expression of the housekeeping gene *GAPDH-2 *was used to standardize the amount of input cDNA in each reaction (primers: 5'-ATCGGACGCTTGGTTCTC-3', 5'-TACGGTGCCCTTGAAACG-3'). Once the *GAPDH-2 *CP was determined for each cDNA sample, it was used to normalize all other genes tested from the same sample. *GAPDH-2 *was chosen for normalization because this gene exhibited high levels of expression but no evidence of biased expression among the selected line microarrays (average ± SE log-ratio = -0.09 ± 0.06) or between males and females across the developmental stages used in constructing the EST libraries (results not shown). Calculation of fold increase and decrease in expression of target genes relative to expression levels of *GAPDH *was accomplished using the 2^-ΔΔCT ^method [72]. Four biological replicates were used for each selected line.

## Authors' contributions

RHB developed the EST library, conducted most of the analyses and drafted the ms. XW dissected discs and isolated RNA for microarray analysis and conducted qrtPCR. JM wrote scripts to perform the alignments and estimate relative gene divergence. JLB supervised development and sequencing of the EST libraries. GSW provided guidance throughout the project, analyzed the microarray and qrtPCR data and edited the ms. All authors read and approved the final manuscript.

## Supplementary Material

Additional file 1 **EST Assemblies**. Nucleotide sequence, EST reads and Genbank accession numbers are provided for 11,545 consensus sequences (conseqs). Click here for file

Additional file 2 **EST Annotation**. Detailed annotation of 7,066 cluster sequences. Information is provided for the top hit for each cluster blasted against the protein databases of *Drosophila melanogaster*, *Anopheles gambiae *and the *D. melanogaster *Transposable Element database. Click here for file

Additional file 3 **EST dipteran alignments**. Amino acid alignment files for *Teleopsis dalmanni *EST translations and proteins for *A. gambiae*, *D. melanogaster*, *D. pseudoobscura and D. virilis*. Click here for file

Additional file 4 **Relative rate of protein evolution in *Teleopsis dalmanni***. Branch length (BL) data for each branch of the ML tree is provided. Columns with species abbreviations indicate the BL for branches leading to terminal taxa. The 'Dm-Dp' column indicates the BL for the node uniting *D. melanogaster *and *D. pseudoobscura *and the 'Dros' column indicates the BL for the node uniting *D. melanogaster*, *D. pseudoobscura and D. virilis*. Click here for file

Additional file 5 **Selection line gene expression values**. Normalized intensity values and log ratios are presented for each microarray oligonucleotide probe for all eight replicate hybridizations. Click here for file
